# Supplementary material for: Molecular-scale visualization of sarcomere contraction within native cardiomyocytes
Source: Nat Commun. 2021 Jul 2;12:4086. doi: 10.1038/s41467-021-24049-0 (PMC8253822; doi:10.1038/s41467-021-24049-0)
Supplement: Supplementary file 1 — Supplementary Information [file 41467_2021_24049_MOESM1_ESM.pdf]

Supplementary Information

Molecular-scale visualization of sarcomere contraction within native cardiomyocytes

Laura Burbaum†, Jonathan Schneider†, Sarah Scholze, Ralph T Böttcher, Wolfgang Baumeister, Petra Schwille, Jürgen M Plitzko, Marion Jasnin*

† These authors contributed equally to this work

* Correspondence to: jasnin@biochem.mpg.de (M.J.)

This PDF file includes:

Supplementary Figures 1-9

Supplementary Tables 1-2

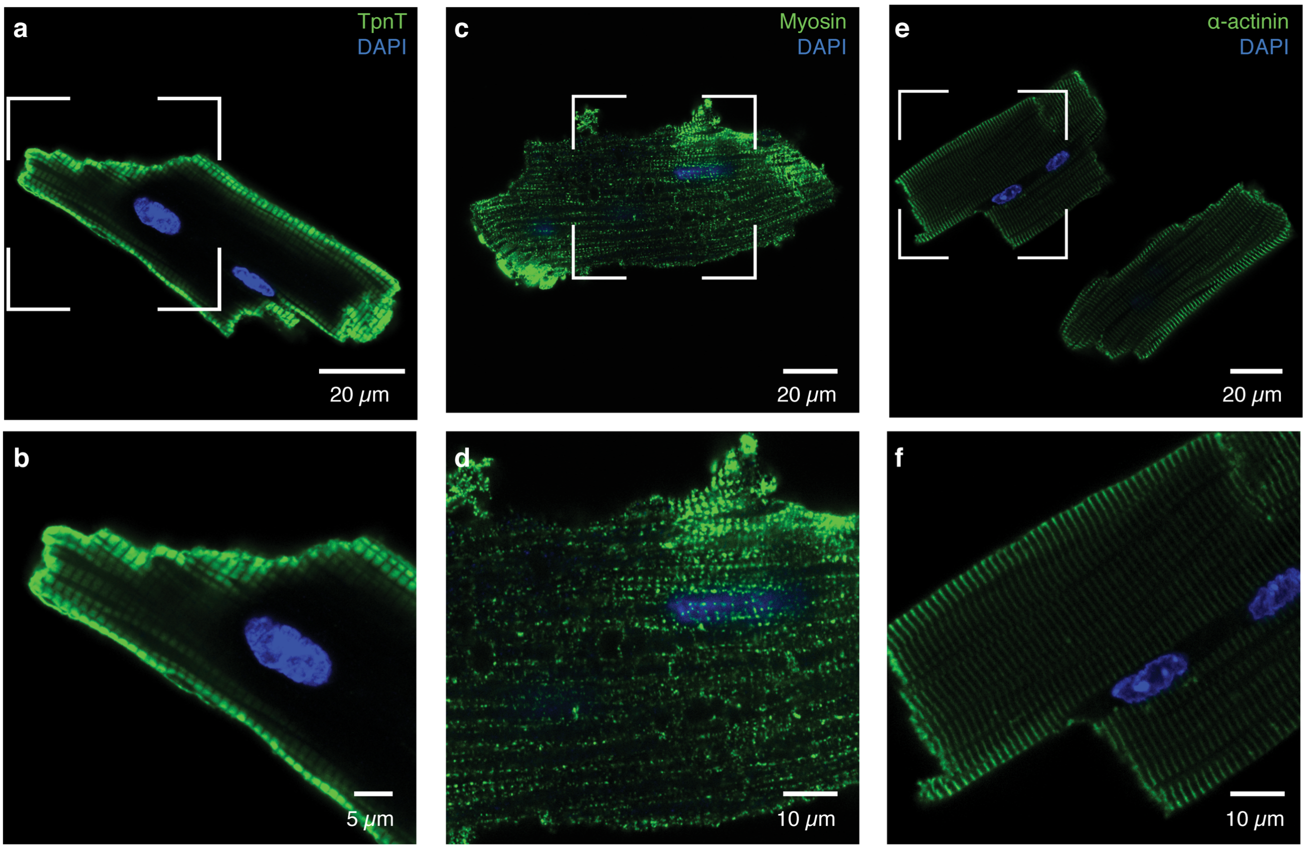

Supplementary Fig. 1 | Myofibrillar organization in adult mouse cardiomyocytes at the microscale. a-f The cells were labelled with antibodies specific for troponin T (TpnT, **a**, **b**), the heavy chain of myosin (Myosin, **c**, **d**) and α-actinin (**e**, **f**), respectively. Thin (**a**, **b**) and thick (**c**, **d**) filaments of adult mouse cardiomyocytes assemble into myofibrils, which are compactly arranged along the main axis of the rod-shaped cells. Myofibrils display regularly spaced Z-disks (**e**, **f**) with a sarcomere length of 1.5 ± 0.1 μm. This sarcomere length is not directly comparable with that of the neonatal cardiomyocytes since cells were isolated from different species. **b**,**d**,**f** Zoomed-in views of the framed regions in **a**, **c**, **e**, respectively. Each experiment was repeated independently at least 3 times with similar results.

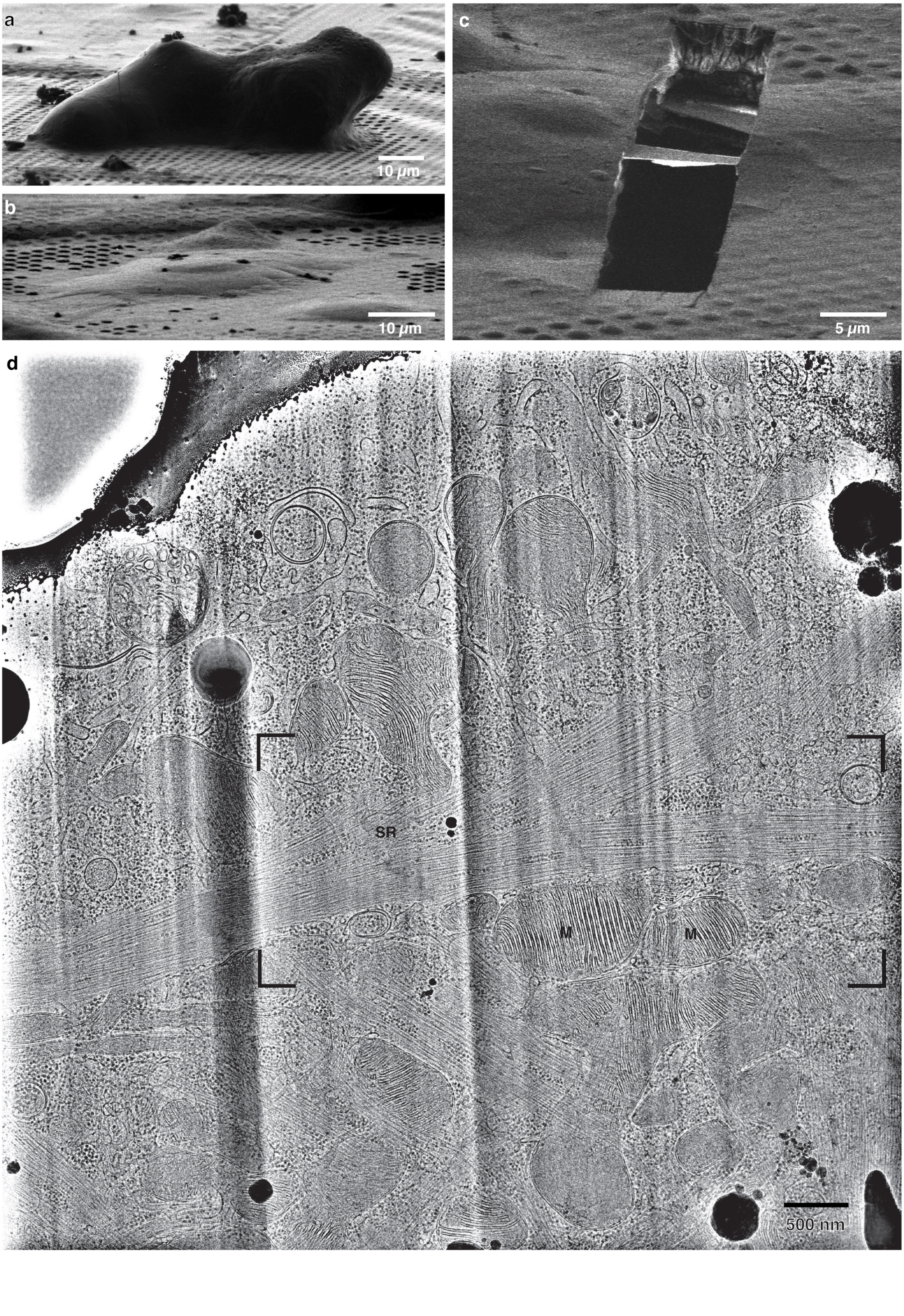
a
10 μm
b
10 μm
c
5 μm
d
SR
M
M
500 nm

Supplementary Fig. 2 | Cryo-FIB sample preparation of neonatal rat cardiomyocytes for cryo-ET imaging. a Scanning electron microscope (SEM) image of a frozen-hydrated adult mouse cardiomyocyte on an EM grid. Vitrification by plunge-freezing is not suitable for these cells because their thickness is larger than 10 µm. **b** Neonatal rat cardiomyocytes are thin, allowing optimal vitrification by plunge-freezing without the need for cryoprotectant. **c** 200-nm-thick lamella prepared in a plunge-frozen neonatal rat cardiomyocyte and imaged by FIB-induced secondary electrons. **d** Full TEM image of a neonatal cardiomyocyte lamella containing the cropped part shown in Fig. 2a. SR, sarcoplasmic reticulum; M, mitochondrion. A total of 8 lamellas prepared from 8 cells was used in this study. Each cell comes from 2 separate experiments in which 4 organisms were mixed and can therefore be considered as a biological replicate. Representative images are shown.

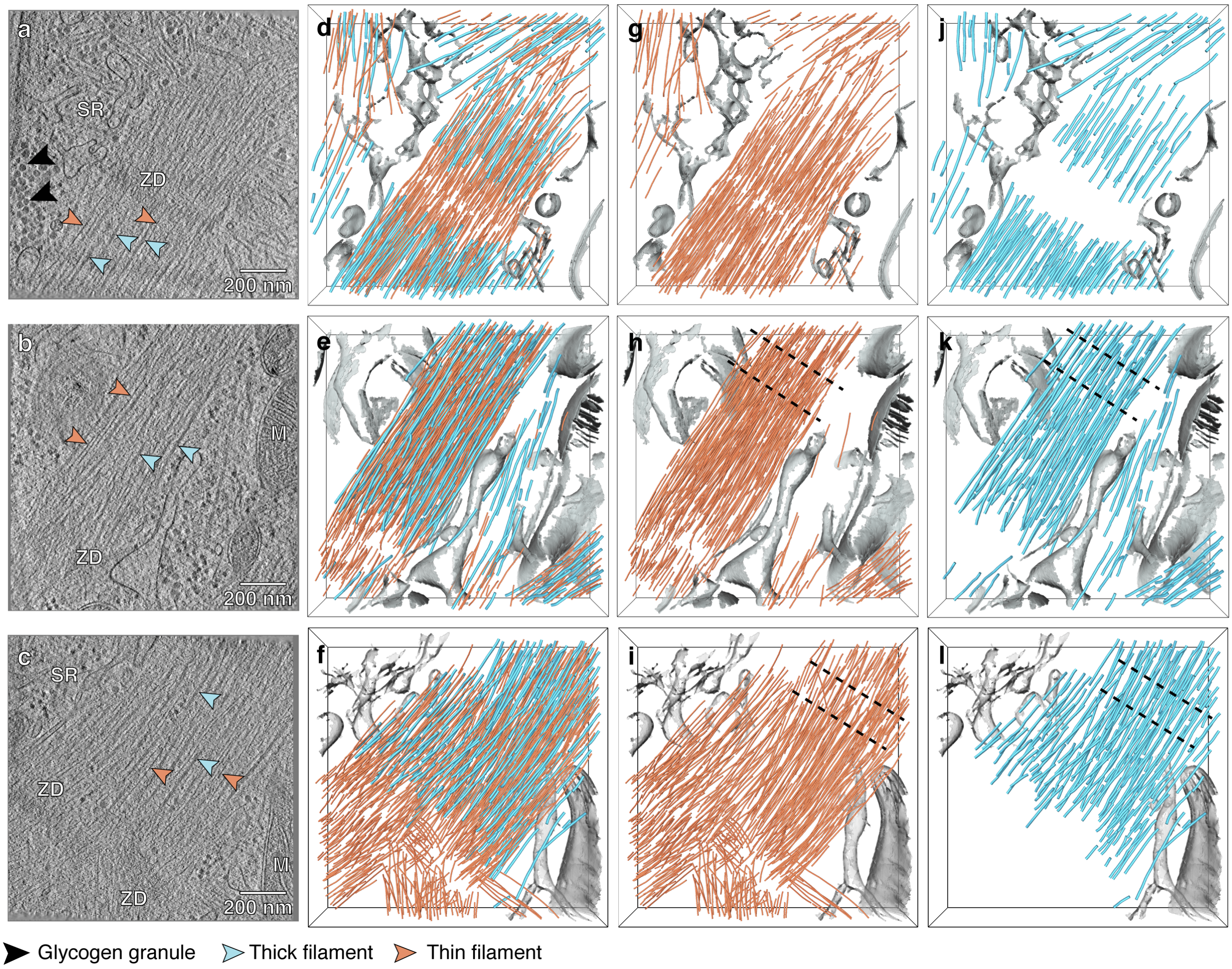

Supplementary Fig. 3 | Nanoscale sarcomere organization revealed by *in situ* cryo-ET. a-c 6.84 nm-thick slices from defocused tomographic volumes acquired in 3 different frozen-hydrated neonatal rat cardiomyocytes, revealing the myofibrillar environment. ZD, Z-disk; M, mitochondrion; SR, sarcoplasmic reticulum. **d-l** Corresponding 3D segmentation of the cellular volumes, showing the nanoscale organization of the thin (orange; **d-i**) and thick (cyan; **d-f**, **j-l**) filaments within unperturbed sarcomeres. Putative myosin bare zones estimated for an average sarcomere length of 1.8 µm are delineated by dashed black lines in **h**, **i**, **k**, **l**. A total of 13 tomograms acquired from 8 cells were used in this study (Supplementary Table 1). Each cell comes from 2 separate experiments in which 4 organisms were mixed and can therefore be considered as a biological replicate. Representative images are shown.

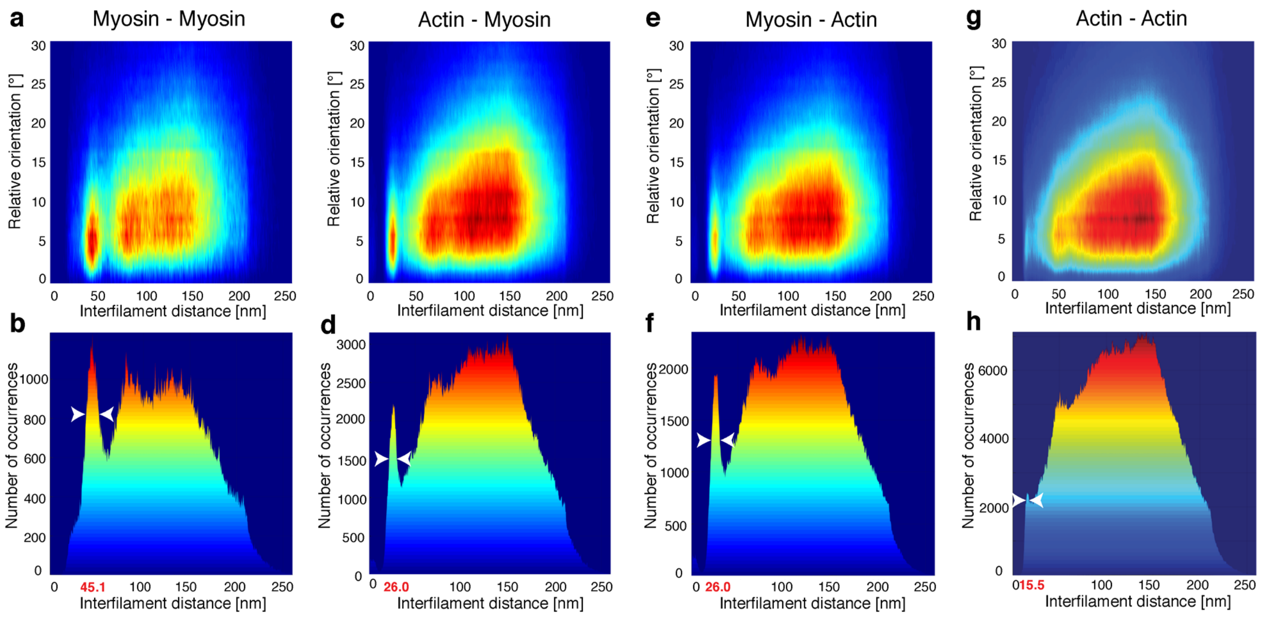

Supplementary Fig. 4 | Near-neighbor analysis of myofilaments in neonatal rat cardiomyocytes. a,**c**,**e**,**g** 2D histogram of interfilament distances and relative orientations between thick filaments (Myosin; **a**), between thick and thin filaments (Actin; **c**, **e**) and between thin filaments (**g**); **b**,**d**,**f**,**h** the corresponding histograms showing the number of occurrences of the interfilament distances. For each point along a filament, the closest point of each neighboring filament is characterized by its distance, d, and relative orientation, θ, with respect to the reference point. The occurrences of (d, θ) are represented in these 2D histograms, for filaments of the same type (that is, only thick filaments (**a**) or only thin filaments (**g**)) as well as for filaments of one type relative to the other (that is, neighboring thick filaments for the thin filaments (**c**), and reciprocally (**e**)). The peak occurring at the shortest interfilament distances and small θ (below 10°) in **a**, **c**, **e**, **g** corresponds to the contribution of the nearest parallel filaments. White arrowheads in **b**, **d**, **f**, **h** indicate the distance range for nearest parallel filaments, obtained from the distances with a number of occurrences higher than two third of the peak maximum. Thick filaments have an interfilament spacing of about 45.1 nm (**a**, **b**) and are found at about 26.0 nm from the thin filaments (**c**-**f**). Thin filaments have their nearest actin neighbors at about 15.5 nm (**g**, **h**).

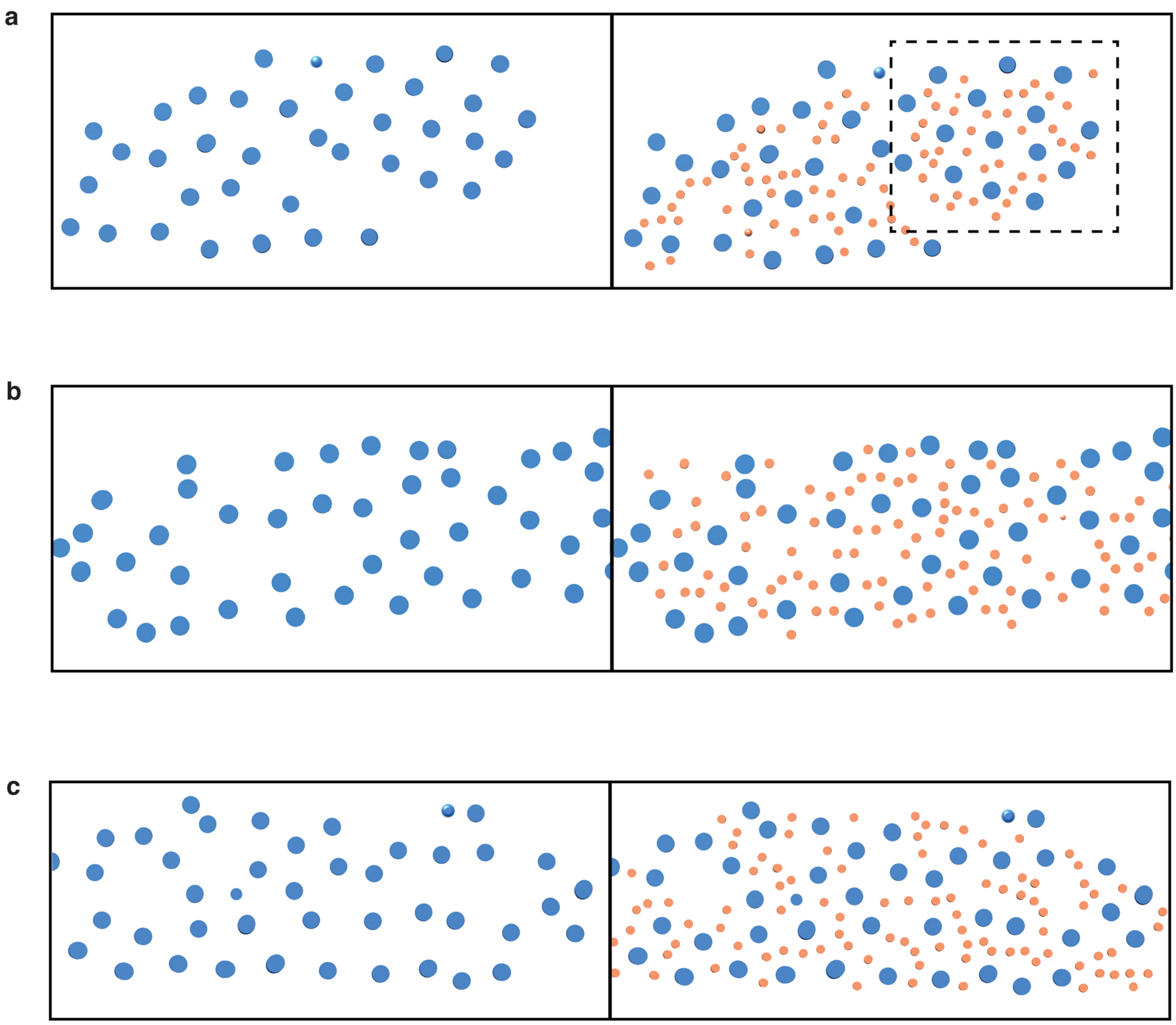

Supplementary Fig. 5 | Examples of cross-sections through myofibrils of neonatal rat cardiomyocytes in the single-overlap region of the A-band. a-c Thick filaments (blue) are hexagonally packed and surrounded by thin filaments (orange, right) at the trigonal positions of the lattice and outside of them. The framed area in (**a**) is shown in Fig. 3i. The slices were obtained from the myofibrils shown in Supplementary Fig. 3e, k (**a**), Fig. 2e, g (**b**) and Supplementary Fig. 3f, l (**c**).

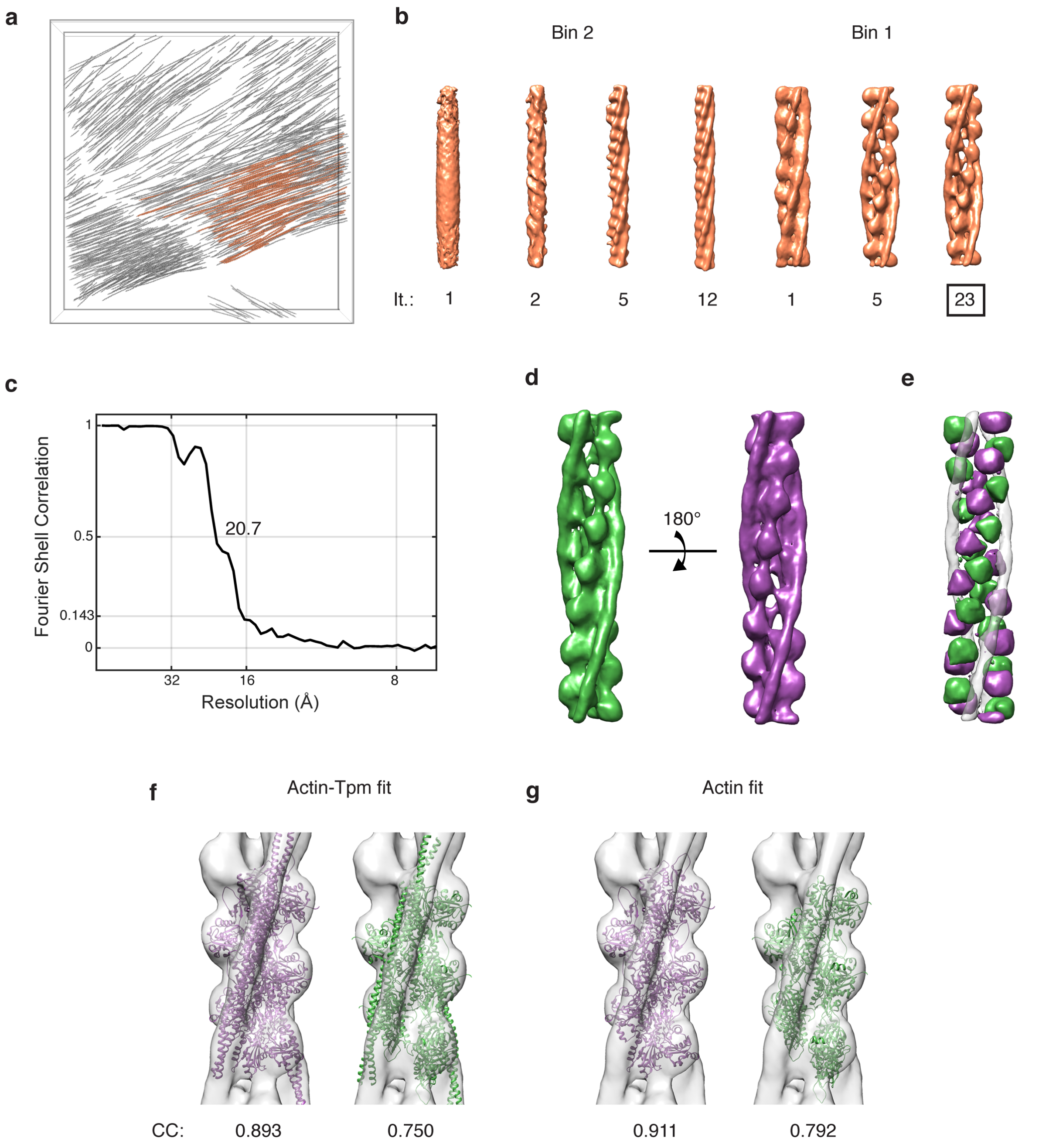

Supplementary Fig. 6 | *De novo* structure generation from cryo-ET data of cardiac thin filaments. a Thin filaments (orange) from the tomogram shown in Fig. 2b and Supplementary Movie 2 used for the generation of a *de novo* reference. **b** Iterative alignment using 2x binned (Bin 2) and unbinned (Bin 1) data resulted in the emergence of the helical pattern of the thin filament and yielded a structure of F-actin in complex with Tpm resolved at 20.7 Å. It, Iteration number. **c** Corresponding Fourier shell correlation plot. Since the data was not processed using gold-standard refinement, a cutoff of 0.5 was used. **d** Filament structures in opposite orientations (green and purple, respectively) and aligned on the Tpm densities. **e** Respective difference maps with superimposed Tpm densities (grey) revealing the actin positions associated to each polarity with respect to the Tpm. **f**, **g** Both orientations (same color code as in **d**) of the pseudoatomic model of the F-actin-Tpm complex (pdb 5jlh) docked into the *in situ* map (grey) shown in purple in **d** (**f**), and corresponding fits using the actin densities only (**g**). The cross-correlation (CC) results permitted to allocate the barbed (+) and pointed (-) end of the *de novo* F-actin structure (Fig. 4a).

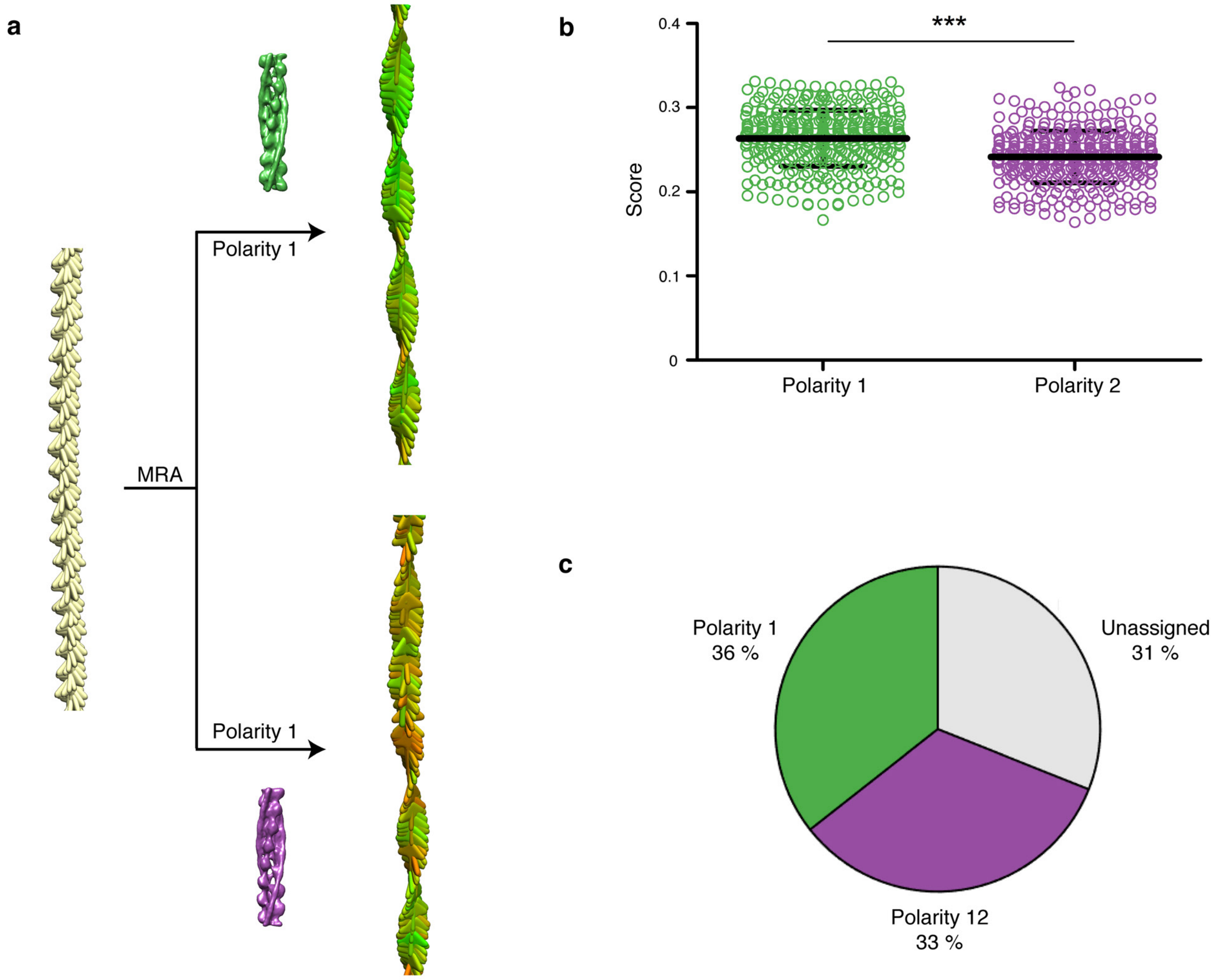

Supplementary Fig. 7 | Workflow for the polarity assessment of the thin filaments imaged by cryo-ET. a Multireference alignment (MRA) procedure applied to one filament with oversampled positions aligned along the *z*-axis and in-plane randomization represented by yellow arrows (left). Each position is aligned independently against a reference of each polarity (in green and purple, respectively), resulting in refined orientations and scores (high scores, green; low scores, red; right). Refinement against the green structure (Polarity 1) leads to uniform helical pattern, whereas alignment against the purple structure (Polarity 2) yields irregularities in the helical pattern. **b** Corresponding scores obtained for the single filament with n=333 independent sampling points for each polarity, with the mean and standard deviation indicated. Unpaired, 2-sided Student's t-test revealed significant differences between both polarities (***: $p < 0.001$). **c** Pie chart representing the results of the polarity assignment for the entire dataset of 594,317 subvolumes with $p < 0.05$. Source data are provided as a Source Data file.

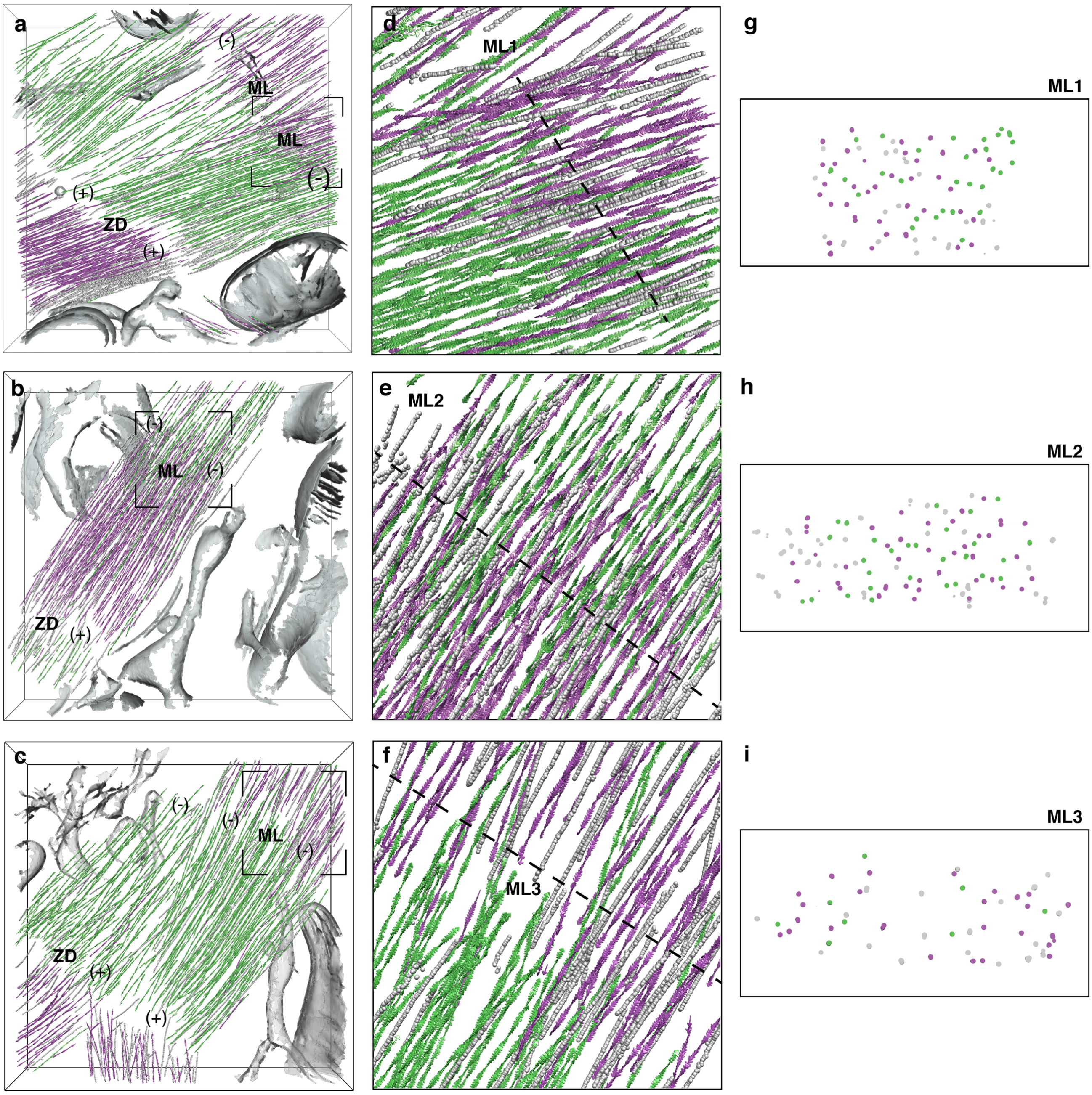

Supplementary Fig. 8 | Polarity assignment and packing of neonatal cardiac thin filaments at the center of the sarcomere. a-c Same examples as in Fig. 4 showing the unassigned filaments in grey, which correspond to 23% of the subvolumes in these datasets. Thin filaments with assigned polarity are represented by colored arrows pointing toward the pointed (-) end. ZD, Z-disk; ML, M-line. **d-f** Zoomed-in views of the framed regions in **a-c**, respectively, showing the thin filament orientations in the framed M-lines (ML1-3). **g-i** Cross-sections through ML1-3 at the locations indicated by the dotted lines in **d-f**.

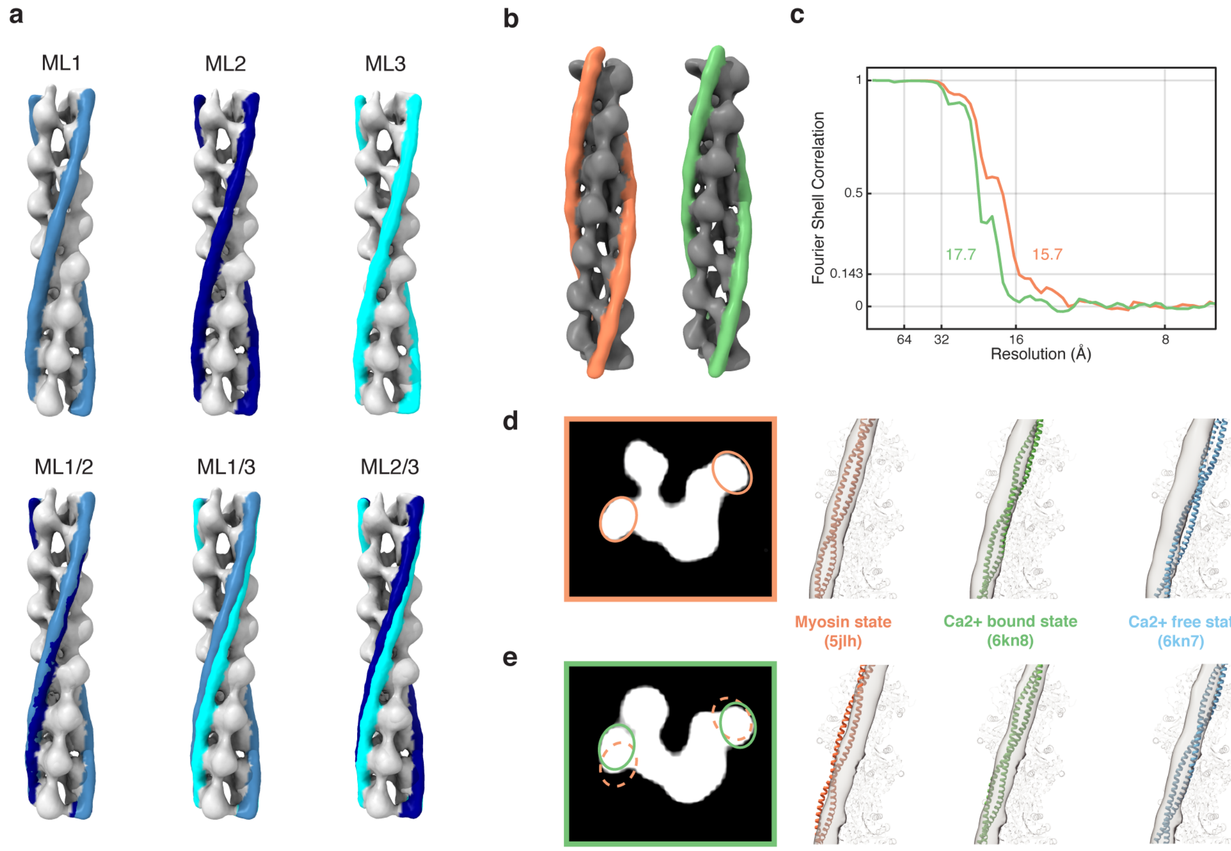

Supplementary Fig. 9 | Resolution and functional state of the native cardiac thin filament structures. a Subtomogram averages of the cardiac thin filament obtained from the data shown in Fig. 4b-d (ML1-3; blue, dark blue and cyan Tpm densities, respectively). Superposing ML1 and ML2 (ML1/2) reveals that these structures are similar. Superposing ML1 or ML2 with ML3 (ML1/3 and ML2/3, respectively) shows that the Tpm density is azimuthally shifted in ML3. **b** Final subtomogram averages associated with the wide (orange Tpm) and narrow (green Tpm) overlap at the M-lines. **c** Gold-standard FSC plots of the subtomogram averages shown in **b** with curves of the same color as the Tpm densities. **d-e** *x-y* slices through the 15.7 Å (**d**) and 17.7 Å (**e**) resolution structures shown in **b**, in which the Tpm densities are highlighted in orange and green, respectively, and close up into the fits of the pseudoatomic models in different states into the maps.

Supplementary Table 1 | Data acquisition parameters

	Volta Phase Plate	Defocus
Microscope	Titan Krios G1	
Magnification	42,000	
Voltage (kV)	300	
Electron exposure (e^-/Å^2)	~100-120	
Detector	Gatan K2 Summit	
Energy filter	Yes	
Slit width (eV)	20	
Phase plate	Yes	No
Defocus range (µm)	-0.5	-3.25 to -5
Tilt range (min/max, starting angle, step)	-50/70°, 10°, 2°	
Tilt scheme	Unidirectional	Dose-symmetric
Pixel size (Å)	3.42	
Tomograms (no.)	3	10
Cells (no.)	1	7
Tomograms (no.) used for packing analysis/Cells (no.)	3/1	6/4
Tomograms (no.) used for averaging and polarity assessment/Cells (no.)	-	10/7

Supplementary Table 2 | Data processing parameters

	Cardiac actin-Tpm filament in the myosin state (EMD-11826)	Cardiac actin-Tpm filament in an intermediate state (EMD-11825)
Pixel size (Å)	3.42	3.42
Subtomograms (no.)	56,858	27,030
Tomograms (no.)/Cells (no.)	2/2	1/1
Symmetry imposed	C1	C1
FSC threshold	0.143	0.143
Map resolution (Å)	15.7	17.7
