# Supplementary material for: Molecular-scale visualization of sarcomere contraction within native cardiomyocytes
Source: Nat Commun. 2021 Jul 2;12:4086. doi: 10.1038/s41467-021-24049-0 (PMC8253822; doi:10.1038/s41467-021-24049-0)
Supplement: Supplementary file 3 — Description of Additional Supplementary Files [file 41467_2021_24049_MOESM3_ESM.pdf]

Description of Additional Supplementary Files

File Name: Supplementary Movie 1
Description: **Neonatal rat cardiomyocytes imaged by bright field.** Cells exhibit irregular, star-like morphology and spontaneous rhythmic contractions.

File Name: Supplementary Movie 2
Description: **Tomogram of the myofibril shown in Figure 2b followed by segmentation of the tomogram.** Thick filaments are in cyan, thin filaments in orange and membranes in grey. Successive rotations of the segmented volume allow to visualize the packing of the myofilaments as well as the Z-disk and I-band regions in 3D.

File Name: Supplementary Movie 3
Description: **Polarity assignment for the neonatal cardiac thin filaments shown in Figure 2e, f and Supplementary Movie 2.** Thin filaments are originally shown in orange and then colored according to the assigned polarity (Fig. 4a). Sampled positions correspond to individual actin monomers represented by arrows pointing toward the pointed (-) end, which follow the in-plane angle retrieved from subtomogram averaging. This representation allows to observe the twist of individual thin filaments. The movie zooms successively on the orientation of a single filament, and on the barbed ends of the filaments of adjoining bundles converging to the Z-disk shown in Fig. 4b, e.

File Name: Supplementary Movie 4
Description: **Polarity assignment for the neonatal cardiac thin filaments shown in Supplementary Figure 3e, h and f, i.** Thin filaments are represented by arrows pointing toward the pointed end and colored according to the assigned polarity. The arrows follow the in-plane angle derived from subtomogram averaging. First half highlights the wide overlap between thin filaments of opposite polarity at the M-line shown in Fig. 4c (ML2). Second half shows the M-line in Fig. 4d (ML3) where thin filaments barely overlap.

File Name: Supplementary Movie 5
Description: **In situ subtomogram average of the neonatal cardiac thin filament in the myosin state.** The structure resolved at 15.7 Å consists of a central actin filament (red and yellow) surrounded by 2 Tpm strands (orange). A close-up shows the fit of a pseudoatomic model of the F-actin-Tpm complex in the myosin state (pdb 5jlh) into the central part of the structure, highlighting successively the fit in the central actin monomer and in one of the Tpm strands.
