# Supplementary material for: Molecular-scale visualization of sarcomere contraction within native cardiomyocytes
Source: Nat Commun. 2021 Jul 2;12:4086. doi: 10.1038/s41467-021-24049-0 (PMC8253822; doi:10.1038/s41467-021-24049-0)
Supplement: Supplementary file 5 — Reporting Summary [file 41467_2021_24049_MOESM5_ESM.pdf]

nature research

Corresponding author(s): Marion Jasnin

Last updated by author(s): May 6, 2021

Reporting Summary

Nature Research wishes to improve the reproducibility of the work that we publish. This form provides structure for consistency and transparency in reporting. For further information on Nature Research policies, see our Editorial Policies and the Editorial Policy Checklist.

Statistics

For all statistical analyses, confirm that the following items are present in the figure legend, table legend, main text, or Methods section.

n/a	Confirmed	
☐	☒	The exact sample size (*n*) for each experimental group/condition, given as a discrete number and unit of measurement
☐	☒	A statement on whether measurements were taken from distinct samples or whether the same sample was measured repeatedly
☐	☒	The statistical test(s) used AND whether they are one- or two-sided<br>*Only common tests should be described solely by name; describe more complex techniques in the Methods section.*
☒	☐	A description of all covariates tested
☒	☐	A description of any assumptions or corrections, such as tests of normality and adjustment for multiple comparisons
☐	☒	A full description of the statistical parameters including central tendency (e.g. means) or other basic estimates (e.g. regression coefficient) AND variation (e.g. standard deviation) or associated estimates of uncertainty (e.g. confidence intervals)
☐	☒	For null hypothesis testing, the test statistic (e.g. *F*, *t*, *r*) with confidence intervals, effect sizes, degrees of freedom and *P* value noted<br>*Give P values as exact values whenever suitable.*
☒	☐	For Bayesian analysis, information on the choice of priors and Markov chain Monte Carlo settings
☒	☐	For hierarchical and complex designs, identification of the appropriate level for tests and full reporting of outcomes
☒	☐	Estimates of effect sizes (e.g. Cohen's *d*, Pearson's *r*), indicating how they were calculated

Our web collection on statistics for biologists contains articles on many of the points above.

Software and code

Policy information about availability of computer code

Data collection	SerialEM 3.7.0
Data analysis	ImageJ 1.52p<br>TOMOMAN (https://github.com/williamnwan/TOMOMAN)<br>MotionCor2 1.2.1<br>IMOD 4.10.18<br>tom_deconv (https://github.com/dtegunov/tom_deconv)<br>CTFFIND 4.1.5<br>NovaCTF (https://github.com/turonova/novaCTF)<br>tomosegmemtv (https://github.com/anmartinezs/pyseg_system/tree/master/code/tomosegmemtv)<br>Amira 2019.2 with XTracing extension<br>MATLAB R2015b<br>TOM Toolbox 1.0<br>STOPGAP 0.7.0 (https://github.com/williamnwan/STOPGAP)<br>UCSF Chimera 1.10.2 with Place Object plugin<br>UCSF ChimeraX 0.9

For manuscripts utilizing custom algorithms or software that are central to the research but not yet described in published literature, software must be made available to editors and reviewers. We strongly encourage code deposition in a community repository (e.g. GitHub). See the Nature Research guidelines for submitting code & software for further information.

Data

Policy information about availability of data

All manuscripts must include a data availability statement. This statement should provide the following information, where applicable:

- Accession codes, unique identifiers, or web links for publicly available datasets
- A list of figures that have associated raw data
- A description of any restrictions on data availability

Data supporting the finding of this manuscript are available from the corresponding author upon reasonable request.
One representative tomogram has been deposited in the EMDB (https://www.ebi.ac.uk/pdbe/emdb) under accession code EMD-12572.
The in situ subtomogram averages of the sarcomeric actin-Tpm filament from neonatal Wistar rat cardiomyocytes have been deposited under accession codes EMD-11826 (for the myosin state) and EMD-11825 (for the intermediate state).
Source data are provided with this paper.

Field-specific reporting

Please select the one below that is the best fit for your research. If you are not sure, read the appropriate sections before making your selection.

☒ Life sciences ☐ Behavioural & social sciences ☐ Ecological, evolutionary & environmental sciences

For a reference copy of the document with all sections, see nature.com/documents/nr-reporting-summary-flat.pdf

Life sciences study design

All studies must disclose on these points even when the disclosure is negative.

Sample size	The sample size (13 tomograms from 8 cells) was chosen because of the practical limitations of the methodology used. It was sufficient to interpret the data in a robust manner according to previous publications (see for example Table S2 in Watanabe et al. Cell 2020 or Supplementary Table 1 in Kiesel et al. Nat Struct Mol Biol 2020).
Data exclusions	The 3 VPP tomograms were excluded from the subtomogram averaging/polarity assessment analyses because the resolutions obtained for the actin-Tpm filament structure for these tomograms were not sufficient.
Replication	A minimum of 3 biological replicates were performed for the light microscopy experiments. All attempts at replication were successful. Electron microscopy data are from 2 isolations of 4 hearts each, with a total of 8 cells investigated. Each cell can therefore be considered as a biological replicate.
Randomization	Neonatal rats (or adult mice) were randomly selected for isolation of ventricular cardiomyocytes. Cells were randomly selected for light microscopy and EM experiments.
Blinding	Blinding is not relevant for this study because it is not a comparative study. Nevertheless, for each isolation, ventricles from 4 hearts were mixed allowing for blinded cell selection.

Reporting for specific materials, systems and methods

We require information from authors about some types of materials, experimental systems and methods used in many studies. Here, indicate whether each material, system or method listed is relevant to your study. If you are not sure if a list item applies to your research, read the appropriate section before selecting a response.

Materials & experimental systems

n/a	Involved in the study	
☐	☒	Antibodies
☐	☒	Eukaryotic cell lines
☒	☐	Palaeontology and archaeology
☐	☒	Animals and other organisms
☒	☐	Human research participants
☒	☐	Clinical data
☒	☐	Dual use research of concern

Methods

n/a	Involved in the study	
☒	☐	ChIP-seq
☒	☐	Flow cytometry
☒	☐	MRI-based neuroimaging

Antibodies

Antibodies used	Monoclonal antibodies against heavy chain cardiac myosin (Abcam, #ab50967, 1:100), α-actinin (Sigma-Aldrich, #A7811, 1:200) and cTnT (Thermo Fisher Scientific, #MA5-12960, 1:200) were used.<br>The anti-mouse secondary antibody (Alexa Fluor™ 488 goat IgG (H+L)) was purchased from Thermo Fisher Scientific (#A11029,

	1:600).
Validation	The anti-heavy chain cardiac myosin antibody has been validated for WB in mice and rats and has been used for IF in human and mouse cardiac muscle cells (https://www.abcam.com/heavy-chain-cardiac-myosin-antibody-ba-g5-ab50967). The anti-α-actinin antibody has been validated for IF in mouse and rat cultured muscle cells (https://www.sigmaaldrich.com/catalog/product/sigma/a7811). The anti-cTnT antibody has been validated for IF in mouse heart tissue and cultured primary cardiomyocytes (https://www.thermofisher.com/antibody/product/Cardiac-Troponin-T-Antibody-clone-13-11-Monoclonal/MA5-12960).

Eukaryotic cell lines

Policy information about cell lines

Cell line source(s)	Neonatal rat ventricular cardiomyocytes were isolated from 3-day-old (P3) wild-type Wistar rats.<br>Adult mouse ventricular cardiomyocytes were isolated from 12-to 15-week-old wild-type C57BL/6 mice.
Authentication	none
Mycoplasma contamination	Cells were imaged by fluorescence and electron microscopy. Cells were either stained with DAPI and examined with a 40x objective, or imaged in the FIB/SEM and TEM with magnifications up to 42,000x. No contamination was found.
Commonly misidentified lines (See ICLAC register)	None

Animals and other organisms

Policy information about studies involving animals; ARRIVE guidelines recommended for reporting animal research

Laboratory animals	P3 wild-type Wistar rats of both sexes. 12-to 15-week-old wild-type C57BL/6 male mice.
Wild animals	none
Field-collected samples	none
Ethics oversight	Housing and use of laboratory animals at the Max Planck Institute of Biochemistry are fully compliant with all applicable German (e.g. German Animal Welfare Act) and EU (e.g. Annex III of Directive 2010/63/EU on the protection of animals used for scientific purposes) laws and regulations concerning care and use of laboratory animals. All of the animals were handled according to approved license (No.5.1-568- rural districts office).

Note that full information on the approval of the study protocol must also be provided in the manuscript.
